# The effect of Virtual Reality on evoked potentials following painful electrical stimuli and subjective pain

**DOI:** 10.1038/s41598-020-66035-4

**Published:** 2020-06-03

**Authors:** E. J. Lier, J. M. Oosterman, R. Assmann, M. de Vries, H. van Goor

**Affiliations:** 1 Department of Surgery, Radboud university medical center Nijmegen, Nijmegen, The Netherlands; 2 Radboud University, Donders Institute for Brain, Cognition and Behaviour, Nijmegen, The Netherlands

**Keywords:** Pain management, Pain

## Abstract

Background: Virtual reality (VR) has been shown to reduce pain, however outcome parameters of previous studies have primarily been of a subjective nature and susceptible to bias. This study investigated the effect of VR on cortical processing of evoked potentials (EPs) and subjectively reported pain. Additionally, we explored whether subjects’ demographic and personal characteristics modulated the effect of VR analgesia. Methods: Three VR conditions were compared in a randomized cross-over study of 30 healthy volunteers: Passive VR (i.e. no interaction possible with the virtual world), active VR (interactive virtual environment) and no VR (black screen). Subjects received noxious electrical stimuli at random intervals during all conditions. EPs, recorded at Cz, were extracted time locked to stimuli. Pain scores were reported after each condition. Results: Active VR significantly decreased pain scores and amplitudes of N1 and P3. Passive VR had no analgesic effect. Age was significantly correlated to pain scores, with older subjects demonstrating larger effects of VR. Gender, game experience, and susceptibility for immersion, did not influence VR analgesia. Conclusion: Active VR decreases pre-perceptual and perceptual brain activity following painful electrical stimuli, corresponding with reduced pain experience. VR has potential to serve as a non-pharmacologic treatment for pain, particularly in elderly patients.

## Introduction

Inadequate pain treatment is a significant health care concern with widespread morbidity^1^. Commonly used analgesics are associated with undesirable side effects and do not always provide sufficient pain relief; many carry additional risks of dependence or addiction^2–4^. Since the subjective experience of pain is influenced by various psychological factors, an increasing number of non-pharmacological alternatives for pain modulation, that affect these psychological factors, has been investigated, including innovative emerging technologies such as Virtual Reality (VR)^5,6^. VR, which completely immerses users in a three-dimensional computer-simulated environment, is thought to reduce pain primarily by distracting attention away from noxious stimuli^6–10^. The distraction hypothesis also suggests that when more attention is required to experience the VR environment, the reduction in pain will be greater^6,11^. In addition to VR content created for the purpose of distraction, the number of VR applications with integrated psychological interventions, such as virtual body ownership or virtual mirror therapy, is increasing. However, the main focus of this study is VR distraction analgesia. VR has shown promising results in reducing pain, anxiety and stress during painful procedures, including during wound dressing changes in burn wound patients and chronic wound patients, dental procedures, and venepuncture^12–16^. Most studies to date have reported an analgesic effect of VR when compared with standard care (no distraction) during painful procedures. VR has additionally been shown to reduce pain in non-procedural acute pain conditions such as postoperative pain^17,18^, in chronic pain conditions like fibromyalgia and musculoskeletal pain^19^, and in experimental pain studies^20,21^. An important limitation in previous research, however, has been a lack of blinding combined with subjective outcomes, introducing a high risk of ascertainment bias^22,23^. Only a small minority of studies to date have studied the phenomenon of VR-assisted analgesia through the use of objective outcome measures like functional Magnetic Resonance Imaging (fMRI) or vital signs as surrogate pain parameters^17,24–26^. In the present study, we used electroencephalography (EEG) to investigate whether VR modulates neural processing of painful stimuli. EEG enables the objective measurement of cortical pain processing by registering evoked potentials (EPs) following painful electrical stimuli. EPs are voltage changes in the EEG that occur within a specified time after a stimulus. By averaging repeated responses, spontaneous signals can be averaged out^27^. This results in a characteristic pattern with peaks of stimuli-related brain activity, which can be subdivided into early and late EP components. The early phase is thought to represent the pre-perceptual sensory response, whereas the late phase represents perceptual processing of pain which is modulated by psychological, attentional and emotional evaluations^28–30^. The purpose of this study was to investigate the effect of passive and active VR applications on EPs and subjective pain experience during painful electrical stimuli. Based on the distraction theory^7,8^, which states that VR reduces pain via modulation of attention, we hypothesized primarily that VR would particularly affect the late components of EPs, and secondarily that active VR, i.e. a virtual environment with interactive elements, would provide more pain reduction than passive VR, i.e. no interaction possible with the virtual world, which is supported by previous VR studies reporting an increased effect of interactive VR compared to passive VR^6,11,31,32^. We additionally explored whether subjects’ demographic characteristics influenced the effect of VR, and evaluated the feasibility of EEG measurements during the use of VR.

## Methods

This randomized three-way cross-over study was conducted at the Radboud university medical center located in Nijmegen, the Netherlands. The medical ethics committee (CMO Arnhem-Nijmegen) waived formal evaluation of this pilot study after initial assessment of the study protocol, in accordance with Dutch law. The study was performed according to the principles of the Declaration of Helsinki (2013). All participants provided written informed consent. This research received no specific grant from any funding agency in the public, commercial, or not-for-profit sectors.

### Participants

Eligible volunteers were healthy adults aged 18 years or older. Exclusion criteria were: uncorrected hearing or vision loss, peripheral sensory loss (e.g. due to diabetic neuropathy or stroke), acute or chronic pain at time of the experiment, or intake of pain medication or other sensory altering substances (alcohol, drugs) within 24 hours of engaging in the experiment. Thirty-two subjects were screened, of whom two subjects were excluded due to neuropathy and chronic use of analgesics. Thirty subjects were randomly allocated to one of six groups. One subject (sequence A-P-C) was excluded from statistical analysis because the quality of EEG recordings for this subject were insufficient (see Results: ‘Feasibility of EEG and VR’ below). A flow diagram of subjects and demographics of subjects included in the analyses are presented in Supplementary file 1.

### Intervention

**Figure 1:**
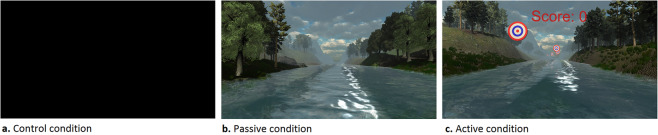
Screenshots of the Virtual Reality conditions: (**a**) control condition, (**b**) passive condition, (**c**) active condition.

Study subjects were exposed to three experimental conditions (counterbalanced): a passive VR condition (P), an active VR condition (A), and a control condition (C). VR was provided through the use of a Head-Mounted Display (HMD, Oculus Rift, consumer version, Menlo Park, United States of America), while the VR applications were developed by our research group (The Virtual River, 2015). Subjects participated in all three conditions, with the condition order determined through randomization using Microsoft Office Excel’s RAND function. Blinding of participants and researchers was not possible due to the nature of the intervention. In the passive VR condition (P), subjects entered a VR environment in which they were riding a boat on a river. Subjects were able to look around in a 360° virtual environment while nature sounds were emitted through headphones. While subjects could alter their view of their virtual environment by moving their head to achieve different vantage points, they were unable to otherwise interact with the VR world in this passive scenario. In the active VR condition (A), an interactive component was added to the same VR environment encountered in the passive condition. Subjects were provided with 120 targets at various points along the river, which they were instructed to hit by aiming their heads in the direction of the target and pressing a button on the key board to “shoot” a ball. Subjects were challenged to hit as many targets as possible. A continuous high score counter was shown to increase subjects’ motivation to score as many points as possible. Every target hit earned ten points, while every shot taken cost two points. In the control condition (C), subjects wore the same HMD but without connection to the computer. Subjects in this scenario observed a dark screen, and no sound was emitted through the headphones. Subjects were subjected to each of the VR conditions for the same length of time. Screenshots from each of the VR conditions are shown in Fig. 1.

### Study procedure

Before entering the VR environment, subjects were asked to complete a questionnaire on personal characteristics and prior game experience (hours per week), and a questionnaire including queries regarding creativity, visualisation, and dreaming (“Creativity Questionnaire”)^33^. Subsequently, the concentric electrode for electrical stimulation, and the EEG cap were installed (details described below). The HMD was carefully placed over the EEG cap and each subject then underwent three VR conditions (order randomized). After each VR condition, subjects reported their average experienced pain score during the electrical stimuli, as measured on a numeric rating scale (NRS) with 1 representing no pain and 10 unbearable pain. Subjects additionally answered a post-exposure questionnaire about their perceived level of immersion in the VR experience^34^. Adverse events were recorded.

### Electrical stimulation

A concentric surface was placed 10 cm from the cubital fossa on the ventral side of the participant’s non-dominant forearm. This concentric electrode (20mm^2^) activated mainly nociceptive A-delta fibers producing a pinprick-like pain sensation that is typical for these superficial nerve endings in the epidermis^35^. Electrical stimuli were provided by a DS7A Constant Current Stimulator (Digitimer Ltd., Welwyn Garden City, Herts, UK). A two-step baseline pain measurement was performed to determine the pain threshold. Electrical stimulation was initiated at 1 mA and was increased by 1 mA for each consecutive stimulus; during a second measure, the stimulation was increased by random increments for each consecutive stimulus. In both measurements, each participant indicated when the stimulus was similar to a pinprick (unpleasant, slightly painful). The stimulus intensity used during the VR conditions was 150% of the average of both pain thresholds, to ensure that the stimuli were painful (mean intensity 15.6 mA ± 5.9). During each study condition, a total of 45 stimuli were given with a random interstimulus interval of five to ten seconds. Subjects were informed that they would receive electrical stimuli but were not informed about the electrical stimulation protocol.

### EEG analysis

EEG was recorded using a multi-channel ActiCAP system (BrainVision, Brain Products GmbH, Germany) of 32 active electrodes according to the international 10–20 system^36^. The ground electrode was placed at the forehead, and the reference electrode in FCz position. Two vertical and two horizontal leads were placed to track eye movements (EOG channels). EEG recordings were analysed offline using BrainVision Analyzer 2.0 (Brain Products GmbH, Germany). First, the sampling rate was downsized from 2000 Hz to 500 Hz. Re-referencing of EEG was necessary since the recording reference (FCz) was next to the point of interest (Cz). Pz was chosen as new reference electrode. For one participant, the mean of P3 and P4 was selected as the reference, due to a bad signal-to-noise ratio of Pz in this subject. Subsequently, ocular correction was applied according to the Gratton and Coles method using the electromyography recordings derived from the EOG channels^37^. A butterfly filter was applied with a low cut-off frequency of 3 Hz and a high cut-off frequency of 30 Hz. Then, EEG recordings were segmented into −100 to 1000 ms epochs time locked to stimulus onset. Baseline correction (−100–0 ms) and artefact rejection were performed automatically by individual channel and checked afterwards. Epochs were rejected from further analysis if data exceeded an amplitude range of −100 µV to 100 µV, if data exceeded the maximal allowed voltage step of 50 µV, or if the lowest allowed activity was lower than 0.5 µV. The maximum percentage of rejected epochs at Cz was 15% in all conditions. Remaining epochs were averaged, and grand averages were calculated per condition. Two peak amplitudes were identified: a negative peak at 130 ms and a positive peak at 238 ms. N1 and P3 peaks were detected in the individual EEG recordings, with N1 defined as the largest negative amplitude value between 80 and 170 ms, and P3 as the largest positive value between 170 and 280 ms after stimulus, based on the grand average peaks per subject. Peak amplitudes and accompanying latencies at Cz were exported per subject and condition.

### Statistical analysis

Data analysis was performed using IBM SPSS statistics v22.0. Descriptive statistics were obtained and analysed for baseline characteristics and interventional effects. All continuous data were normally distributed. Repeated measures analyses of variance (RM-ANOVA) were performed on N1 amplitudes, P3 amplitudes, and pain scores to test differences between VR interventions. Condition (control condition, passive VR, active VR) was the within-subjects variable and EP amplitudes or pain scores were the dependent variables. As we were not able to perfectly balance the order of the conditions encountered by subjects, condition sequence was incorporated as a between-subjects variable. Exploratory post hoc analyses, with Bonferroni correction for multiple comparisons, were performed in the RM-ANOVA. Delta thresholds of outcome variables were calculated for each VR intervention (i.e. Δ Passive VR = passive VR minus control, Δ Active VR = active VR minus control and Δ Active-Passive VR = active VR minus passive VR). Statistical tests were two-tailed and a *p-*value ≤0.05 was considered to be statistically significant. Pearson’s correlation tests were performed to examine correlations between the delta thresholds and personal characteristics gathered from the questionnaires. The influence of gender was analysed using independent T-tests on the delta thresholds. Bonferroni correction was applied for multiple comparisons (15 comparisons), therefore p-values ≤0.003 were considered significant.

## Results

### VR and EPs

**Figure 2:**
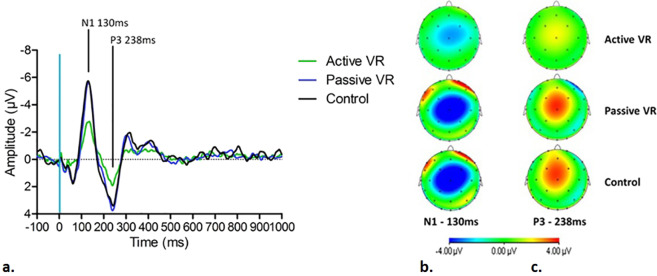
(**a**) Grand average N1 and P3 peaks during active VR, passive VR and the control condition. (**b**) Brain activity topography at 130 ms during each condition. (**c**) Brain activity topography at 238 ms during each condition.

**Figure 3:**
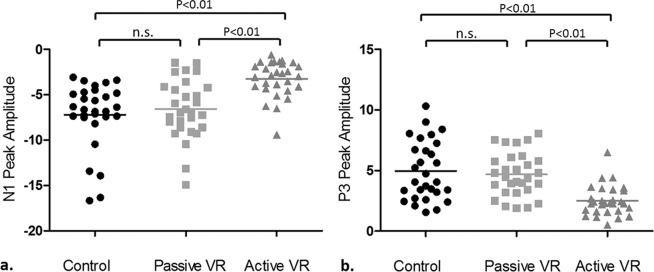
Individual N1 (**a**) and P3 (**b**) amplitudes during each condition.

Grand averages of N1 and P3 amplitudes by VR condition and corresponding brain activity topographies at 130 ms and 238 ms are shown in Fig. 2 (2a–2c respectively). Table 1 summarizes mean values of individual peak amplitudes of N1 and P3 within the window defined earlier. The distributions of N1 and P3 amplitudes in each VR condition are shown in Fig. 3 (3a and 3b, respectively). Maximal brain activity changes were recorded at Cz. A significant effect of VR was found for N1 peak amplitudes (F (2, 46) = 31.218, *p* < *0.01*) and P3 peak amplitudes (F (1.482, 34.079) = 27.217, *p* < *0.01*). Post hoc analysis revealed that active VR resulted in significant amplitude reduction of N1 and P3 peaks, compared to the control condition (N1 *p* < *0.01, P3 p* < *0.01)* and passive VR (N1 *p* < *0.01, P3 p* < *0.01)*. No significant differences were found between passive VR and the control condition. N1 and P3 effects were not influenced by the sequence of conditions.

**Table 1:** Mean individual peak amplitudes of N1 and P3, and pain scores during each condition.

	Control Condition	Passive VR	Active VR
N1 Amplitude (µV, mean ± sd)	−7.21 ± 3.65	−6.58 ± 3.26	−3.24 ± 1.97
P3 Amplitude (µV, mean ± sd)	4.97 ± 2.44	4.69 ± 1.81	2.51 ± 1.24
Pain score (NRS, mean ± sd)	5.59 ± 1.35	4.93 ± 1.53	3.17 ± 1.54

### VR and pain scores

**Figure 4:**
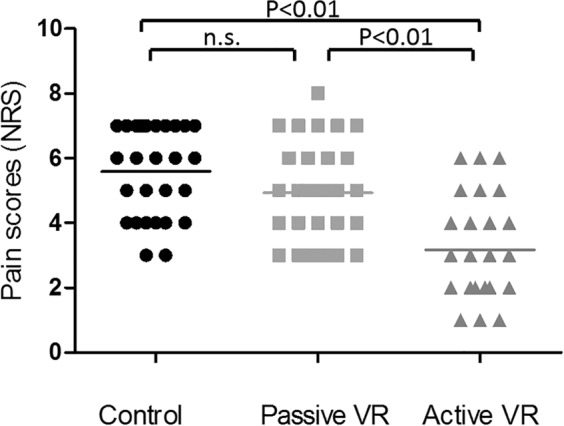
Individual pain scores (NRS) during each condition.

A significant effect of VR was found for subjectively reported pain scores (F (2, 46) = 39.069, *p* < *0.01*). Post hoc analysis revealed that active VR resulted in lower pain scores compared to the control condition (*p* < *0.01)* and the passive condition (*p* < *0.01)*. Although mean pain scores were lower during passive VR compared to the control condition, no significant differences were found. No significant treatment order effect was observed. Individual and mean pain scores per condition are shown in Fig. 4.

### Individual characteristics

Age was significantly correlated to pain scores for Δ passive VR (r = −0.460, *p* < 0.001), with older subjects demonstrating larger VR effects. A similar effect was observed for Δ active VR, but this trend was not significant after Bonferroni correction (*p* = 0.012) (Supplementary file 1, Fig. 2). Neither subjects’ gender nor their prior gaming experience significantly related to the impact of VR on N1 or P3 peak amplitudes, or its effect on pain scores. Individual differences in creativity, visualisation (day)dreaming, and subjects’ reported levels of immersion in the VR worlds were also not significantly correlated with the effect of VR.

### Feasibility of EEG and VR

A reliable EEG signal was acquired during all VR conditions. However, the HMD caused major artefacts in the recording of the frontal, temporal, and occipital EEG leads located underneath the elastic strap belt. There were also artefacts due to movement or blinking, though these did not hinder EP extraction. In one subject, the quality of EEG recordings was very low due to a worse signal-to-noise ratio (>95% removed epochs in all conditions), therefore, this subject was excluded from statistical analyses. Two subjects reported dizziness during the passive VR and active VR condition. After removing the HMD, the dizziness resolved within one minute. These adverse events had no consequences for the study procedure.

## Discussion

To our knowledge, this is the first study investigating the effect of VR applications with varying levels of interactivity, on Evoked Potentials (EPs) and subjectively reported pain. By measuring both subjective and objective outcome measures, we aimed to provide greater insight into modulation of brain activity following painful stimuli as a result of VR analgesia, and to avoid the limitations related to bias which have been encountered in prior non-blinded studies measuring subjective outcomes. We observed significantly reduced pain scores and peak amplitudes of N1 and P3 during active VR, compared to passive VR and the no-VR control scenario. Passive VR had no significant effect on EPs or pain scores. Differences in pain ratings during passive VR scenarios were correlated with age, indicating a larger subjective pain reduction in elderly subjects. Other personal traits did not affect the effect of VR analgesia.

Our observation of reduced amplitudes of late EP-components suggests that active VR alters pain experience through modulation of perceptual pain processing. Since the intensity of the stimulus was equal during all conditions, we can conclude that VR modifies the intensity of the noxious input received from the peripheral nervous system at thalamic and thalamocortical levels, before the signal is detected in the cerebral cortices. These findings support the distraction hypothesis, reinforcing the theory that the thalamus is involved in pain modulation through, among other things, cognitive factors such as attention^38^. Reduced late EP amplitudes have been previously reported in several studies investigating the effect of cognitive and auditory distraction on painful laser-evoked potentials^29,39^. In general, the modulation of these components corresponds with changes in subjective pain ratings. Thus, the modulation of late EP components supports the reliability of observed differences in subjective pain experience during active VR.

VR seems to modulate not only late perceptual processing of pain, but also pre-perceptual brain responses, represented by changes of early peak amplitudes following painful electrical stimuli. The range of ‘early’ amplitudes varies between different types of EPs^40^, however the amplitudes of EPs at 130 ms after the stimulus that were observed in our study were considered to represent early sensory processing, that, among other things corresponded to the parameters of the noxious stimulus. However, it cannot be excluded that an earlier negative wave precedes the N1-P3 complex as demonstrated in laser evoked potentials^40^. This very early component is maximal over the temporal area contralateral to the stimulated side and could not be detected in this study. Since the intensity of the painful stimulus was equal during all conditions, the observed differences in N1 amplitudes between active VR and the control condition cannot be caused by changes in the stimulus. Possibly, perceptual processing of stimuli during distraction starts at an early stage, prior to the cognitive perceptual process, such as, for example, through thalamic filtering or attenuation of stimuli during pre-perceptual processing, as previously suggested by Picton^30^. However, more research is needed to confirm this hypothesis.

Based on the distraction theory, and previous VR studies, we hypothesized that we would observe a larger effect of active VR compared to passive VR, which was indeed found in this study^6,11,31,32^. We also hypothesized that passive VR would have an analgesic effect compared to no VR^41,42^. However, we did not note any significant effect of passive VR distraction on either pain scores or EPs. Some subjects reported that the passive VR world bored them after a few minutes, and some even reported that they were more immersed during the control condition because the black screen encouraged imagination. Therefore, we hypothesize that it is not only the interactive component of the VR content that is of key importance, but also the attention paid to the VR world by the subject, or a combination of both factors that include overlapping aspects, which determines the ultimate analgesic effect of VR. It is possible that the passive VR application used in our study may have lacked other distractive elements present in different passive VR applications used in comparable studies which were found to have a positive analgesic effect^41,42^.

A similar dearth of distractive elements may also explain the differing results seen in a comparable study investigating the effect of visual distraction through VR on EPs and pain scores^43^. In that study, participating healthy volunteers and chronic migraine patients viewed a presentation of virtual images representing either a hospital waiting room or an imaginary waiting room with coastal views. In healthy volunteers, no differences between scenes were found; however, chronic migraine patients reported lower pain scores and demonstrated smaller amplitudes of late EP-components when viewing the imaginary waiting room. One could argue that patients need less distractive elements when presented with a VR environment, however, caution is advised when extrapolating VR results seen in study volunteers to those that might be experienced by actual patients with regard to passive VR in relation to the distraction level of the medium.

The use of EPs offers new insights in VR pain modulation, but this method also has some disadvantages. Reduced brain activity during VR has previously been observed in fMRI studies^24,26^. As a result of the high temporal resolution of EEG, EPs represent a more direct brain response within milliseconds after the painful stimulus, in contrast to fMRI results, that involve a lag time of 3–6 seconds from the inciting incident^44^. Therefore, EPs enable a more precise temporal evaluation of responses, for example assessments of early versus late components of pain processing. However, the high spatial resolution of fMRI enables a more specific topographic mapping of changes in brain activity compared to EEG.

It should be mentioned that the EP that is observed is not nociceptive specific, but reflects an overall response following the noxious stimulation^40^. This response can also be influenced by the saliency of the stimulus, or within condition habituation. To reduce the potential effects of expectancy on EPs in our experiment, the stimuli were given with a random interstimulus interval. Furthermore, no significant order effects were observed for the analyses on N1, P3. Additional testing of within-condition habituation within blocks was not possible, because, in order to improve the signal-to-noise ratio, the total of 45 stimuli were necessary to create a grand average.

There are also practical considerations with regard to the combined use of EEG and VR. The strap belt used in our study resulted in significant artefacts in the EEG signal from leads located underneath the device, including the temporal leads. As a result of these artefacts, re-referencing the recording reference to the average of all electrodes was not possible. This is also the reason why one participant has been re-referenced differently. In our study, maximal brain activity changes of both N1 and P3 amplitudes were recorded at Cz, without a sign of lateralisation, and recordings of this lead were not affected by the VR equipment. For these reasons, we have chosen to analyse both amplitudes at Cz. Secondly, to minimize disruption caused by removal of the HMD between conditions, all conditions were therefore performed in one session, which limited our ability to achieve an appropriate washout time to limit carry-over effects. Nonetheless, we expect that carry-over effects were minimal in our study, since our results were not affected by the sequence of conditions. Finally, the amount of head movement triggered by subjects’ interaction with their VR environment may have varied for each condition, causing differences in EPs between conditions. To minimize the effects of these differences, we analysed the grand averages of EPs, which represent the average of repeated measures of brain activity time-locked directly after the stimulus. Since most of the noise occurs randomly, the influence of spontaneous activity could be most effectively minimized by analysing the grand average of EPs.

Many studies have demonstrated the effectiveness of VR as a treatment for pain, however, studies specifying population characteristics modulating the effects of VR are limited^45^. For this purpose, we explored several personal traits, such as gender, age, previous game experience and immersion. In order to study the influence of ageing on VR, we included a wide age range varying from young adults to persons of a more advanced age, to mirror the predominant age composition of most hospital inpatients. Given the larger analgesic effect noted among elderly volunteers, one could argue that VR may be particularly suitable for treating pain in elderly patients as a non-pharmacological alternative to common painkillers. Older patients, particularly those with comorbidities, are more susceptible to side effects of both opioid and non-opioid analgesics due to changes in pharmacokinetics and pharmacodynamics, and frequent concurrent use of interacting or even counteracting medications^46^. VR was well accepted by our elderly volunteers, which concurs with a recent report showing a positive attitude towards VR after a VR experience and a low rate of adverse effects among elderly people^47^. The other traits we examined did not influence the degree of the subject’s VR pain reduction, suggesting that VR can be ‘prescribed’ for a wide variety of patients. However, since this study describes only a first exploration of traits that might influence VR pain reduction, more trials are needed to investigate whether VR has a larger effect in specific populations.

## Conclusion

Active VR decreases amplitudes of both pre-perceptual components of EPs following painful stimuli, which are related to early modulation of sensory stimuli, and amplitudes of late components of EPs, which are associated with perceptual pain processing through a psychological, emotional and cognitive evaluation of nociceptive stimuli. The modulation of pain processing during VR was also observed through subjects’ reporting of reduced pain scores. Our findings offer important evidence supporting the efficacy of VR analgesia, by demonstrating that active VR significantly reduces subjective pain experience (NRS), and objectively through changes in EPs following painful electrical stimuli. Findings suggest that VR has the potential for use as an effective non-pharmacologic treatment option for pain, particularly in elderly patients.

## Supplementary information

Supplementary information.

Supplementary Figure 2.

Supplementary Figure 1.

## Data Availability

The datasets used and/or analysed during the current study are available from the corresponding author on reasonable request.

## References

[1] Sinatra R (2010). Causes and consequences of inadequate management of acute pain. Pain. Med..

[2] Gan TJ, Habib AS, Miller TE, White W, Apfelbaum JL (2014). Incidence, patient satisfaction, and perceptions of post-surgical pain: results from a US national survey. Curr. Med. Res. Opin..

[3] Benyamin R (2008). Opioid complications and side effects. Pain. Physician.

[4] Sostres C, Gargallo CJ, Arroyo MT, Lanas A (2010). Adverse effects of non-steroidal anti-inflammatory drugs (NSAIDs, aspirin and coxibs) on upper gastrointestinal tract. Best. Pract. Res. Clin. Gastroenterol..

[5] Scheffler M, Koranyi S, Meissner W, Strauss B, Rosendahl J (2018). Efficacy of non-pharmacological interventions for procedural pain relief in adults undergoing burn wound care: A systematic review and meta-analysis of randomized controlled trials. Burns.

[6] Hoffman H. G. *et al*. In *Virtu*al Realit*y for Psychological and Neurocognitive Interventions* (ed Bouchard S. Rizzo A.) Ch. 8, 195–208 (Springer Nature, 2019).

[7] McCaul KD, Malott JM (1984). Distraction and coping with pain. Psychol. Bull..

[8] Melzack R, Wall PD (1965). Pain mechanisms: a new theory. Science.

[9] Triberti S, Repetto C, Riva G (2014). Psychological factors influencing the effectiveness of virtual reality-based analgesia: a systematic review. Cyberpsychol Behav. Soc. Netw..

[10] Hoffman HG, Doctor JN, Patterson DR, Carrougher GJ, Furness TA (2000). Virtual reality as an adjunctive pain control during burn wound care in adolescent patients. Pain.

[11] Gutierrez-Maldonado J, Gutierrez-Martinez O, Cabas-Hoyos K (2011). Interactive and passive virtual reality distraction: effects on presence and pain intensity. Stud. Health Technol. Inf..

[12] Alshatrat, S. M., Alotaibi, R., Sirois, M. & Malkawi, Z. The use of immersive virtual reality for pain control during periodontal scaling and root planing procedures in dental hygiene clinic. *Int J Dent Hyg* (2018). 10.1111/idh.12366 30216688

[13] Piskorz, J. & Czub, M. Effectiveness of a virtual reality intervention to minimize pediatric stress and pain intensity during venipuncture. *Journal for specialists in pediatric nursing: JSPN* **23** (2018). 10.1111/jspn.12201 29155488

[14] Chan E, Foster S, Sambell R, Leong P (2018). Clinical efficacy of virtual reality for acute procedural pain management: A systematic review and meta-analysis. Plos one.

[15] Luo H, Cao C, Zhong J, Chen J, Cen Y (2019). Adjunctive virtual reality for procedural pain management of burn patients during dressing change or physical therapy: A systematic review and meta-analysis of randomized controlled trials. Wound Repair. Regen..

[16] Malloy KM, Milling LS (2010). The effectiveness of virtual reality distraction for pain reduction: a systematic review. Clin. Psychol. Rev..

[17] Mosso-Vazquez JL, Gao K, Wiederhold BK, Wiederhold MD (2014). Virtual reality for pain management in cardiac surgery. Cyberpsychol Behav. Soc. Netw..

[18] Tashjian VC (2017). Virtual Reality for Management of Pain in Hospitalized Patients: Results of a Controlled Trial. JMIR Ment. Health.

[19] Jones T, Moore T, Choo J (2016). The Impact of Virtual Reality on Chronic Pain. Plos one.

[20] Demeter N, Josman N, Eisenberg E, Pud D (2015). Who can benefit from virtual reality to reduce experimental pain? A crossover study in healthy subjects. Eur. J. pain..

[21] Magora F, Cohen S, Shochina M, Dayan E (2006). Virtual reality immersion method of distraction to control experimental ischemic pain. Isr. Med. Assoc. J..

[22] Wood L (2008). Empirical evidence of bias in treatment effect estimates in controlled trials with different interventions and outcomes: meta-epidemiological study. BMJ.

[23] Boutron I (2017). CONSORT Statement for Randomized Trials of Nonpharmacologic Treatments: A 2017 Update and a CONSORT Extension for Nonpharmacologic Trial Abstracts. Ann. Intern. Med..

[24] Hoffman Hunter G., Richards Todd L., Van Oostrom Trevor, Coda Barbara A., Jensen Mark P., Blough David K., Sharar Sam R. (2007). The Analgesic Effects of Opioids and Immersive Virtual Reality Distraction: Evidence from Subjective and Functional Brain Imaging Assessments. Anesthesia & Analgesia.

[25] Wiederhold BK, Gao K, Sulea C, Wiederhold MD (2014). Virtual reality as a distraction technique in chronic pain patients. Cyberpsychol Behav. Soc. Netw..

[26] Hoffman HG (2004). Modulation of thermal pain-related brain activity with virtual reality: evidence from fMRI. Neuroreport.

[27] Luck, S. J. *An introduction to the event-related potential technique*. (MIT, 2005).

[28] Lee MC, Mouraux A, Iannetti GD (2009). Characterizing the cortical activity through which pain emerges from nociception. J. neuroscience: Off. J. Soc. Neurosci..

[29] Garcia-Larrea L, Peyron R, Laurent B, Mauguiere F (1997). Association and dissociation between laser-evoked potentials and pain perception. Neuroreport.

[30] Picton, T. W. *Human event-related potentials*. (Elsevier, 1988).

[31] Wender R (2009). Interactivity Influences the Magnitude of Virtual Reality Analgesia. J. Cyber Ther. Rehabil..

[32] Dahlquist LM (2007). Active and passive distraction using a head-mounted display helmet: effects on cold pressor pain in children. Health Psychol..

[33] Lier EJ, Harder J, Oosterman JM, de Vries M, van Goor H (2018). Modulation of tactile perception by Virtual Reality distraction: The role of individual and VR-related factors. Plos one.

[34] Schubert T (2003). The sense of presence in virtual environments: A three-component scale measuring spatial presence, involvement, and realness. Z. für Medienpsychologie.

[35] Katsarava Z (2006). A novel method of eliciting pain-related potentials by transcutaneous electrical stimulation. Headache.

[36] Klem GH, Luders HO, Jasper HH, Elger C (1999). The ten-twenty electrode system of the International Federation. The International Federation of Clinical Neurophysiology. Electroencephalogr. Clin. Neurophysiol. Suppl..

[37] Gratton G, Coles MG, Donchin E (1983). A new method for off-line removal of ocular artifact. Electroencephalogr. Clin. Neurophysiol..

[38] Ab Aziz CB, Ahmad AH (2006). The role of the thalamus in modulating pain. Malays. J. Med. Sci..

[39] Legrain V, Guerit JM, Bruyer R, Plaghki L (2002). Attentional modulation of the nociceptive processing into the human brain: selective spatial attention, probability of stimulus occurrence, and target detection effects on laser evoked potentials. Pain.

[40] Mouraux A, Iannetti GD (2009). Nociceptive laser-evoked brain potentials do not reflect nociceptive-specific neural activity. J. Neurophysiol..

[41] Dahlquist LM (2009). Effects of videogame distraction using a virtual reality type head-mounted display helmet on cold pressor pain in children. J. Pediatr. Psychol..

[42] Hoffman HG (2006). Virtual reality helmet display quality influences the magnitude of virtual reality analgesia. J. Pain..

[43] de Tommaso M (2013). Virtual visual effect of hospital waiting room on pain modulation in healthy subjects and patients with chronic migraine. Pain. Res. Treat..

[44] Mullinger K, Bowtell R (2011). Combining EEG and fMRI. Methods Mol. Biol..

[45] Indovina P (2018). Virtual Reality as a Distraction Intervention to Relieve Pain and Distress During Medical Procedures: A Comprehensive Literature Review. Clin. J. Pain..

[46] Kaye AD, Baluch A, Scott JT (2010). Pain management in the elderly population: a review. Ochsner J..

[47] Huygelier H, Schraepen B, van Ee R, Vanden Abeele V, Gillebert CR (2019). Acceptance of immersive head-mounted virtual reality in older adults. Sci. Rep..

