# Supplementary material for: The effect of Virtual Reality on evoked potentials following painful electrical stimuli and subjective pain
Source: Sci Rep. 2020 Jun 3;10:9067. doi: 10.1038/s41598-020-66035-4 (PMC7270181; doi:10.1038/s41598-020-66035-4)
Supplement: Supplementary file 1 — Supplementary information. [file 41598_2020_66035_MOESM1_ESM.pdf]

Supplementary file 1

Manuscript: The effect of Virtual Reality on evoked potentials following painful electrical stimuli and subjective pain

E.J. Lier*1, J. M. Oosterman [2], R. Assmann[1], M. de Vries[1], H. van Goor[1]

1. Department of Surgery, Radboud university medical center
Nijmegen, The Netherlands. 2. Radboud University, Donders Institute for Brain, Cognition and Behaviour, Nijmegen, The Netherlands

Table 1. Personal characteristics

**Subject characteristics (N=29)**	
Gender[a]<br>- Male<br>- Female	 18 (62.1%)<br>11 (37.9%)
Age (years)[b]	36.3 (14.4)
Creativity questionnaire[b,c]	37.4 of 50 (3.8)
Previous VR experience[a]<br>- Yes<br>- No	 18 (62.1%)<br>11 (37.9%)
VR experience (hours)[b]	3.1 (9.5)
Level of immersion[b,d]<br>- Passive VR<br>- Active VR	 21.8 (3.8) of 30<br>25.2 (3.2) of 30
a: n(%), b: mean (sd)<br>*c: maximum score 50 indicates high level of creativity, such as using visualisation often in daily life.*<br>*d: maximum score of 30 : maximally immersed in the virtual world.*	

Figure 1. Flow diagram of subjects (Conditions: C=control, P=Passive Virtual Reality, A=Active Virtual Reality)

Figure 2. Correlations of age and Δ pain scores for active VR minus baseline (n.s.), passive VR minus baseline ($r=-0.635$, $p<0.001$) and active VR minus passive VR (n.s.).
