# Supplementary figures and images for: The effect of Virtual Reality on evoked potentials following painful electrical stimuli and subjective pain
Source: Sci Rep. 2020 Jun 3;10:9067. doi: 10.1038/s41598-020-66035-4 (PMC7270181; doi:10.1038/s41598-020-66035-4)

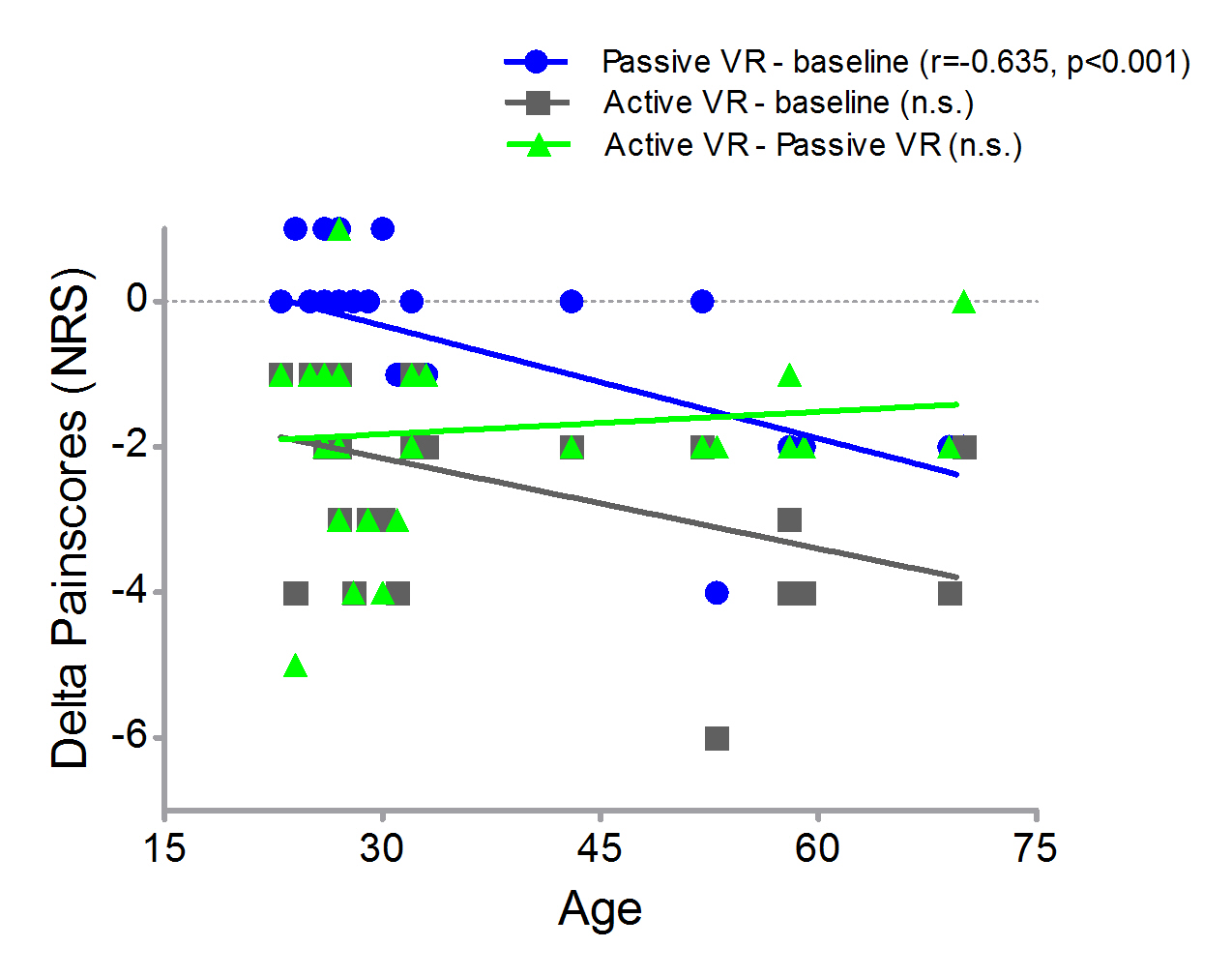

Supplement: Supplementary file 2 — Supplementary Figure 2. [file 41598_2020_66035_MOESM2_ESM.jpg]

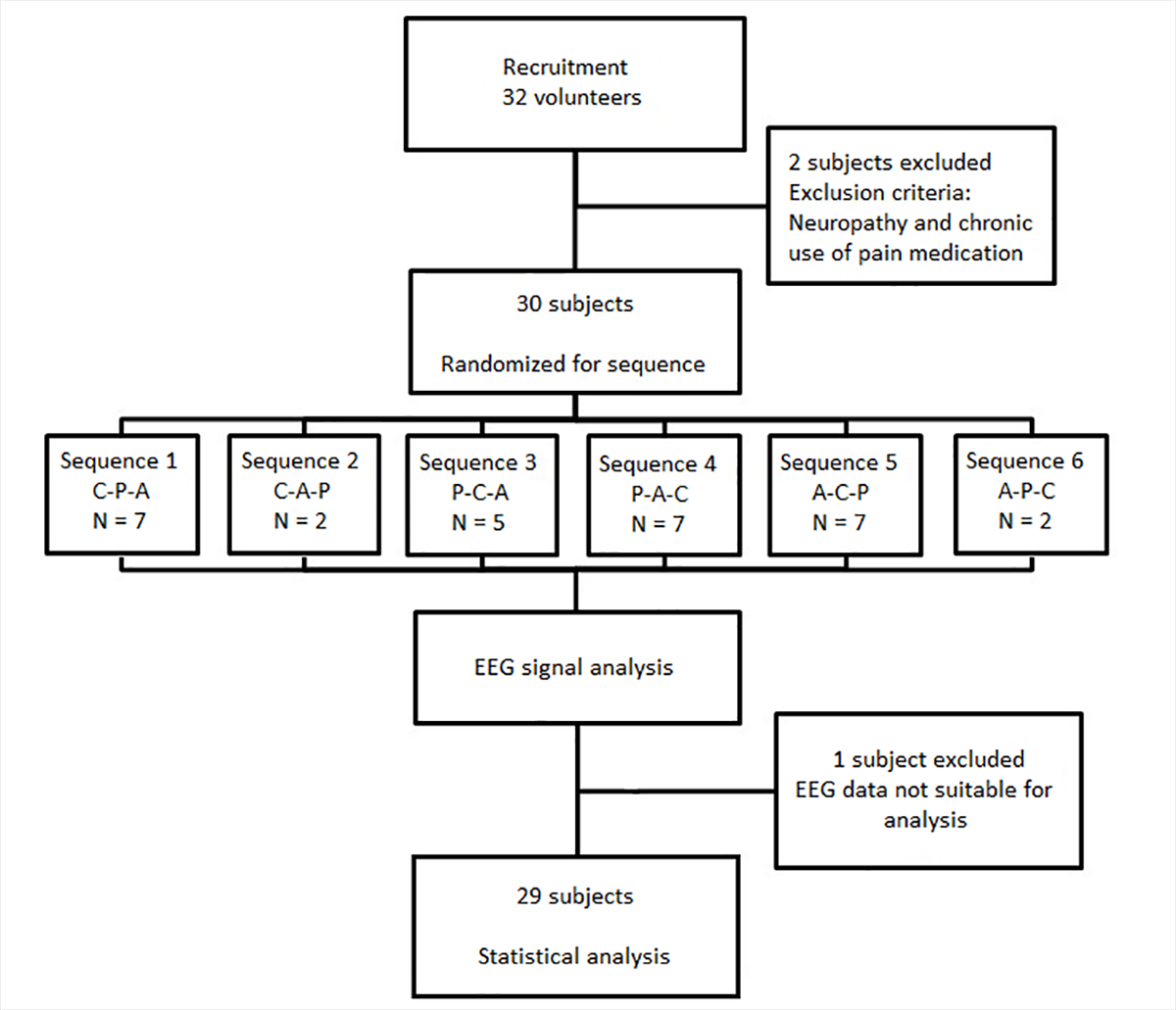

Supplement: Supplementary file 3 — Supplementary Figure 1. [file 41598_2020_66035_MOESM3_ESM.tif]
